# A cross-sectional survey about behavior of wearing condoms among college students who engage in sexual activity: the mediating role of attitudes of preventive behavior

**DOI:** 10.3389/fpubh.2025.1544564

**Published:** 2025-05-23

**Authors:** Li Qi, Ronghuang Guo, Yi Qu, Xiaomei Li

**Affiliations:** ^1^School of Nursing, Health Science Center, Xi’an Jiaotong University, Xi’an, China; ^2^School of Nursing, Qiqihar Medical University, Qiqihar, China; ^3^Department of Neurosurgery, Qiqihar First Hospital, Qiqihar, China

**Keywords:** college students, sexual behaviors, perception of infection risk, perceived disorders of preventive behavior, perceived benefits of preventive behavior, attitudes of preventive behavior, behavior of wearing condoms, HIV

## Abstract

**Background and objectives:**

Although the global human immunodeficiency virus (HIV) infection rate has decreased in recent years, the proportion and number of HIV-infected individuals aged 18–24 years has increased. The main mode of HIV transmission is sexual, a large proportion of the infected population are college students, and numerous health education activities have been implemented to prevent HIV infection, where mastery of relevant knowledge greatly improves. Nevertheless, the effectiveness of necessary protective measures is not ideal during actual behavior, indicating insufficient conversion of prevention knowledge to behavioral. To understand the factors and interactions that influence preventive behavior, we conducted a cross-sectional exploratory study.

**Materials and methods:**

Participants (*n* = 1,111) were students from Heilongjiang Province. A survey questionnaire was distributed through online social platforms. The questionnaire collected data on sociodemographic variables, perception of infection risk (PIR), perceived benefits of preventive behavior (PBPB), perceived disorders of preventive behavior (PDPB), attitudes of preventive behavior (APB), and behavior of wearing condoms (BWC).

**Results:**

BWC did not differ significantly according to gender, academic qualification pursued, or subject major (*p* ≥ 0.05), but did differ significantly based on sexual orientation, receiving HIV infection prevention education, and commercial and casual sexual behaviors. No significant correlation was detected between PIR and APB (*p* ≥ 0.05), while PBPB and APB were significantly positively and PDPB and APB significantly negatively correlated (*p* < 0.05). Further, there was no significant correlation between PBPB and BWC (*p* ≥ 0.05), while PIR and APB were positively correlated with BWC (*p* < 0.05), and there was a negative correlation between PDPB and BWC (*p* < 0.05). APB did not mediate between PDPB and BWC, but APB partially mediated between PDPB and BWC, accounting for approximately 9.96% of the total effect value, and played a fully mediating role (100%) between PBPB and BWC.

**Conclusion:**

Our analysis indicates that PBPB acts on BWC through APB, PIR directly affects BWC, and PDPB can directly affect BWC, while also influencing BWC through APB. Future efforts to increase PBPB, PIR, and APB, reduce PDPB, and promote the use of condoms in sexual behavior among college students are warranted.

## 1 Introduction

Human immunodeficiency virus (HIV) infection continues to impose a heavy burden and remains a major public health issue (1). To date, there is no effective vaccine to prevent (2), and no effective medication to cure HIV (3); however, viral load and disease progression speed can be controlled through medication (4). Hence, prevention remains the most important measure to control the number of HIV infections.

After years of effort, HIV infection in China demonstrates an overall low prevalence trend (5, 6); however, the proportion and numbers of HIV-infected individuals aged 18–24 years has increased, rather than decreasing (7). The affected population includes many college students (8), and there is a risk of further HIV infection spread to the intimate partners of infected individuals, which would inevitably generate public health pressure (9). Pre exposure prevention and post exposure prevention, as effective prevention strategies, can significantly reduce the risk of infection (10–14). Early diagnosis and treatment can help infected individuals receive timely medical intervention and reduce the risk of virus transmission (14). In summary, the comprehensive use of pre-and post exposure prevention, voluntary counseling and testing, risk behavior management, condom use, and early intervention strategies can effectively prevent the spread of HIV (14).

The main route of HIV infection among college students is sexual transmission (15), and a considerable proportion of HIV infection cases are due to individuals not wearing condoms during sexual intercourse (16, 17). Over many years, health education aimed at preventing HIV infection has been vigorously implemented, and these educational activities have achieved significant results in enhancing the knowledge of college students about HIV infection prevention and AIDS-related awareness (18); however, necessary protective measures are often not taken proactively during actual sexual behavior, indicating insufficient conversion of prevention knowledge at the behavioral level (19). This phenomenon prompted us to explore where the “gap” between knowledge dissemination and practical protective behavior arises.

The aim of this study was to establish a theoretical model using structural equation modeling to explain the factors influencing of behavior of wearing condoms (BWC) among college students who engage in sexual activity, as well as the relationships between identified influencing factors. Our research results provide basic data that can improve health education and intervention strategies for preventing HIV infection among college students.

### 1.1 Theoretical model construction and research hypotheses

**Figure 1:**
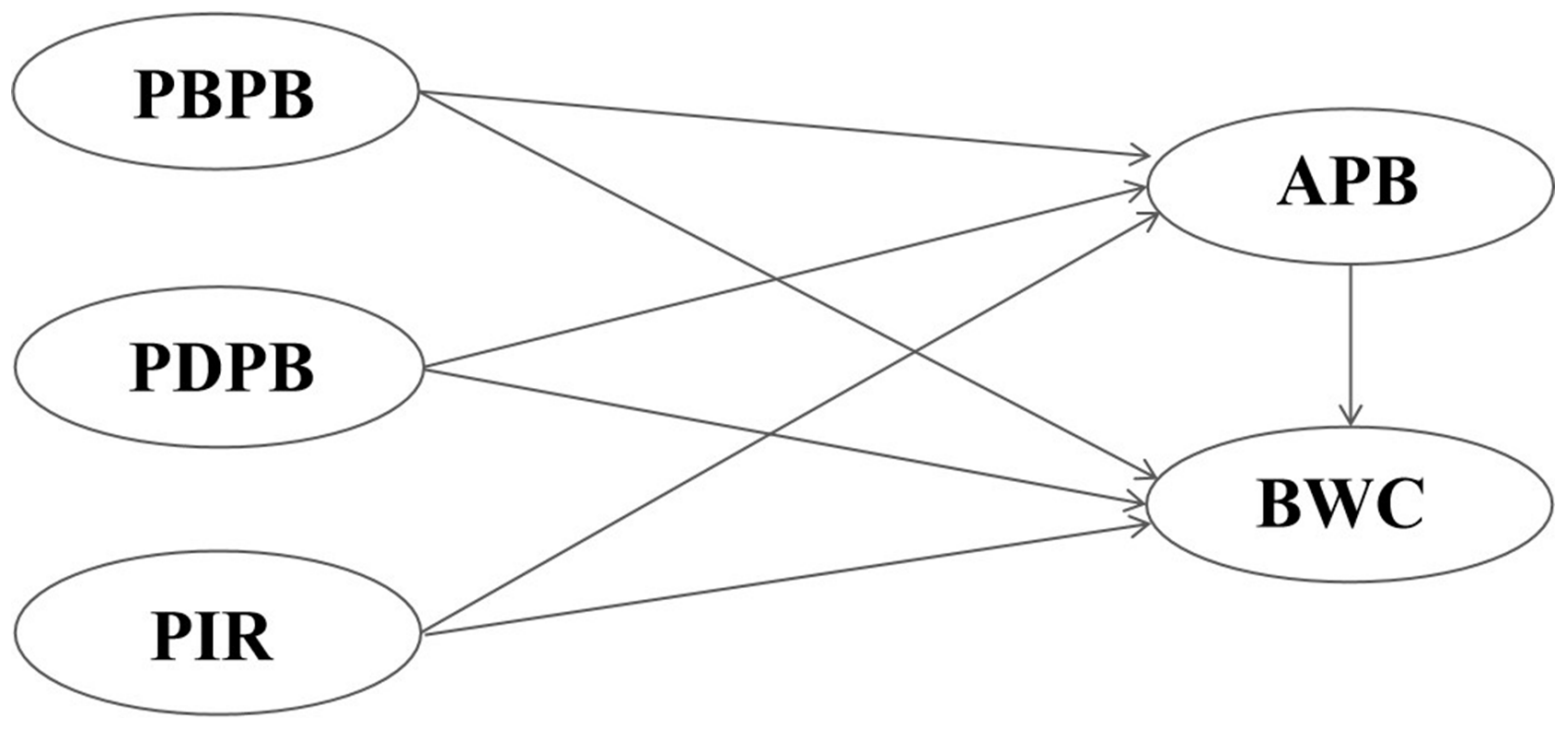
Research framework.

The health belief model (20, 21) is a widely used theory of individual behavior change. Health beliefs (22) also have strong predictive effects on behavior, as follows: First, the perception of diseases, which, in our study, equated to an individual’s perception of possible HIV infection; Second, the perception of preventive behavior, mainly including its benefits during sexual activity (such as using condoms to reduce the risk of HIV transmission) and the perception of obstacles (such as the impact of condoms on sexual pleasure or difficulty in obtaining them), which collectively affect an individual’s attitudes toward preventive behavior. The knowledge, attitude, practice model is a classic theory used to explain and predict healthy behaviors (23). This model suggests that behavior change is a progressive process, from acquiring knowledge to forming attitudes, and then to actual action (24). Attitude is an important intermediate link between knowledge and practical behavior. There are two facets of attitude in this context (25): attitude toward the disease, referring to an individual’s understanding of the severity and potential risks of HIV infection; and attitude toward preventive behavior, referring to an individual’s trust in the effectiveness of preventive measures, such as wearing condoms during sexual activity. Although both facets of attitude have predictive effects on behavior, research suggests that attitudes toward preventive behavior are more important (26). Therefore, in this study we mainly considered attitudes toward preventive behavior. If individuals firmly believe that preventive measures can effectively prevent the spread of HIV, they are more likely to translate them into practical protective actions, thereby reducing the risk of infection. In summary, we explored perception of infection risk (PIR) about HIV, perceived benefits of preventive behavior (PBPB), perceived disorders of preventive behavior (PDPB), and attitudes of preventive behavior (APB), as factors potentially affecting behavior of wearing condoms (BWC); constructed a theoretical model (Figure 1); and tested research hypotheses as follows:

*H1*: PIR exerts a positive effect on APB.

*H2*: PBPB exerts a positive effect on APB.

*H3*: PDPB exerts a negative effect on APB.

*H4*: PIR exerts a positive effect on BWC.

*H5*: PBPB exerts a positive effect on BWC.

*H6*: PDPB exerts a negative effect on BWC.

*H7*: APB exerts a positive effect on BWC.

## 2 Materials and methods

### 2.1 Ethics and consent

This study was approved by the Ethics and Research Review Committee of Qiqihar Medical University in China ([2021]150); the date of approval was 8 November, 2021. All participants in the study provided informed consent.

### 2.2 Study population and design

In China, young students aged 18–24 are mainly college students and junior college students, while considering the impact of medical background on HIV prevention knowledge. This study selected junior college and college students, and who majoring in medicine and non-medical fields as research subjects. For the convenience of questionnaire distribution and data collection, universities in the author’s city of work and nearby cities were selected for the survey.

A cross-sectional anonymous online survey of students aged ≥ 18 years from five universities in Qiqihar, Daqing, Harbin in Heilongjiang Province, who filled out the questionnaire with informed consent and had no reading or questionnaire barriers, was conducted from November 2023 to December 2023. The survey questionnaire was distributed through online social platforms (WeChat and QQ) and automatically collected after completion.

### 2.3 Questionnaire design

The survey questionnaire was proofread and revised by a panel of experts in relevant fields, including disease prevention experts, behavioral psychologists, and statisticians. The first version of the questionnaire was pre surveyed among 30 medical students, and based on the pre survey results, some ambiguous items were modified, resulting in 23 questions. The questionnaire usually took < 10 min to complete.

The questionnaire comprised the following parts: (1) sociodemographic variables, including gender, education level, subject major, sexual orientation, whether received health education to prevent HIV infection, whether engaged in commercial sexual activity, and whether engaged in casual sexual activity; (2) Perception of infection risk (3 items), perceived benefits of preventive behavior (3 items), perceived disorders of preventive behavior (4 items), and attitudes of preventive behavior (5 items); and (3) Behavior of wearing condoms (1 item). Behavior of wearing condoms refers to whether participants have used condoms throughout their most recent sexual intercourse before the survey. Among the sexual transmission routes of HIV, vaginal intercourse and anal intercourse are the main transmission routes (27, 28). Therefore, in this study, sexual intercourse refers to both vaginal and anal intercourse.

This study included perception of infection risk, perceived benefits of preventive behavior, perceived disorders of preventive behavior, attitudes of preventive behavior, and 7 sociodemographic variables, totaling 11 independent variables. The sample size was taken as 10 times the number of independent variables, and considering 20% of invalid questionnaires, at least 138 people need to be included in this study. To ensure the stability and accuracy of structural equation modeling analysis, the sample size should be greater than 200 (29). Based on the above two considerations, the sample size of this study was designed to be at least 200 people.

### 2.4 Variables and measurements

Each item in perception of infection risk, perceived benefits of preventive behavior, perceived disorders of preventive behavior, and attitudes of preventive behavior was scored on a 5-point Likert scale, with a score of 1 indicating strong disagreement and a score of 5 indicating strong agreement. Behavior of wearing condoms was transformed to a binary variable (1 = yes and 0 = no), to facilitate analysis. “yes” indicates that participants had used condoms throughout their most recent sexual intercourse before the survey.“no” means not using or not using condoms throughout the entire process when engaging in sexual activity before the survey.

### 2.5 Questionnaire reliability

In this study, the calculated Cronbach’s alpha and Kaiser-Meyer-Olkin values for the questionnaire were 0.786 and 0.859, respectively, indicating that the instrument is valid and reliable for data-gathering activities (30).

### 2.6 Statistical analysis

The software packages SPSS 21.0 and Analysis of Moment Structures 26.0 were used for statistical analyses. Calculation of descriptive statistics, correlation analysis, and structural equation modeling (SEM) were conducted. The fit of the SEM was tested using chi-square/degrees of freedom (*χ^2^*/DF) and root mean square error of approximation (RMSEA) analyses, where 1 < χ^2^/DF < 3 and RMSEA < 0.05 indicated a better fit. Comparative fit index (CFI), Tucker-Lewis index (TLI), and incremental fit index (IFI) served as incremental fit indices, where CFI > 0.95, TLI > 0.95, and IFI > 0.95 indicated better fit (30). A *p*-value of < 0.05 was considered statistically significant.

## 3 Results

### 3.1 Demographic data and behavior of wearing condoms

Students participating in the pre survey were not included in the final sample. A total of 21,445 questionnaires were collected, among which 1,111 college students reported engaging in sexual activity. Among the 1,111 sexually active individuals mean ± standard deviation age was 19.45 ± 1.309 years, the median age of participants was 19 years (range, 18–25 years), and 613 (55.18%) were male. Further, 292 (26.28%) were pursuing a college degree, 554 (49.86%) were medical majors, 32 (3.15%) had same-sex orientation, 1,032 (92.89%) had heterosexual orientation, 44 (3.96%) had bisexual orientation, 1,017 (91.54%) had received health education related to HIV infection prevention, 26 (2.34%) had undertaken commercial sexual activity, and 107 (9.63%) had engaged in casual sexual activity.

Behavior of wearing condoms did not differ significantly (*p* > 0.05) according to gender, academic qualifications pursued, or subject major; however, behavior of wearing condoms differed significantly (*p* < 0.05) among individuals with differing sexual orientation. The proportion of people with heterosexual orientation who reported wearing condoms during sexual relations was significantly higher than that of people with homosexual orientation (*p* < 0.05), while the differences between the group with bisexual orientation and those with other orientations were not significant (*p* > 0.05). Behavior of wearing condoms differed significantly between respondents who did and did not receive health education related to HIV infection prevention, between those who did and did not report engaging in commercial sexual activity, and between those who did and did not engage in casual sexual activity (*p* < 0.05). See Tables 1, 2 for details.

**Table 1:** Summary statistics of BWC according to participant sociodemographic characterization (*n* = 1,111).

Variable	Group	BWC		*N* (%)	*χ*^2^ (*P*)
		Yes (%)	No (%)		
Gender	Male	506 (82.54)	107 (17.46)	613 (55.18)	1.151 (0.283)
	Female	423 (84.94)	108 (21.69)	498 (44.82)	
Academic qualifications pursued	Junior college degree	683 (83.39)	136 (16.61)	819 (73.72)	0.114 (0.735)
	College degree	246 (84.25)	46 (15.75)	292 (26.28)	
Major	Medical	465 (83.94)	89 (16.06)	554 (49.86)	0.081 (0.776)
	Non-medical	464 (83.30)	93 (16.70)	557 (50.14)	
Sexual orientation	Same-sex	24 (68.57)	11 (31.43)	35 (3.15)	8.751 (0.013*)
	Opposite sex	872 (84.50)	160 (15.50)	1,032 (92.89)	
	Bisexual	33 (75.00)	11 (25.00)	44 (3.96)	
Received health education on HIV infection prevention	Yes	858 (84.37)	159 (15.63)	1,017 (91.54)	4.902 (0.027*)
	No	71 (75.53)	23 (24.47)	94 (8.46)	
Commercial behavior	Yes	15 (57.69)	11 (42.31)	26 (2.34)	13.064 (0.000***)
	No	914 (84.24)	171 (15.76)	1,085 (97.66)	
Temporary behavior	Yes	70 (65.42)	37 (34.58)	107 (9.63)	28.625 (0.000***)
	No	859 (85.56)	145 (14.44)	1,004 (90.37)	

*p < 0.05; ***p < 0.001.

**Table 2:** Cross tabulation of BWC and sexual orientation data (*n* = 1,111).

BWC	Sexual orientation			Total
	Same-sex	Opposite sex	Bisexual	
Yes	24_a_	872_b_	33_a,b_	929
	2.6%	93.9%	3.6%	100.0%
No	11_a_	160_b_	11_a,b_	182
	6.0%	87.9%	6.0%	100.0%
Total	35	1,032	44	1,111
	3.2%	92.9%	4.0%	100.0%

Different subscript letters (a, b) indicate a significant difference between numbers.

### 3.2 SEM fitting index results

Analysis of the fit of the SEM generated in our study demonstrated that *χ*^2^/DF was 2.900, RMSEA was 0.041, CFI was 0.991, TLI was 0.988, and IFI was 0.991, indicating that the overall fit of the research model was acceptable.

### 3.3 Model analysis results

**Figure 2:**
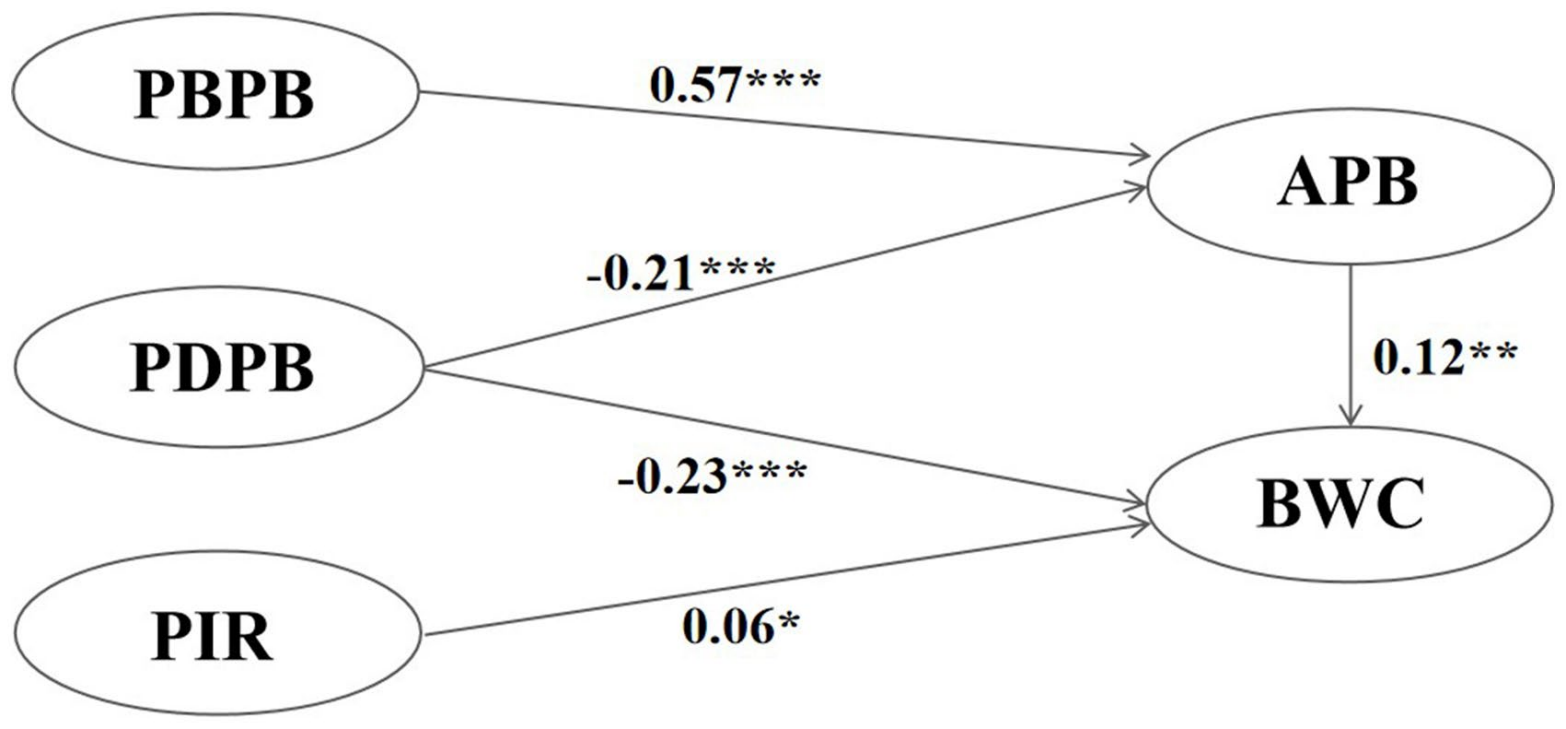
Results after deletion of non-significant paths. *p < 0.05; **p < 0.01;***p < 0.001.

Path analysis demonstrated that perception of infection risk had a significant positive impact on behavior of wearing condoms, supporting H4, but had no effect on attitudes of preventive behavior, which does not support H1. Perceived benefits of preventive behavior had a significant positive effect on attitudes of preventive behavior, supporting H2, but no effect on behavior of wearing condoms, which did not support H5. Perceived disorders of preventive behavior had significant negative impacts on attitudes of preventive behavior and behavior of wearing condoms, supporting H3 and H6. Attitudes of preventive behavior had significant positive impact on behavior of wearing condoms, thus supporting H7. Hypothesis test results are presented in Table 3, and the results of deleting unsupported paths are shown in Figure 2.

**Table 3:** Hypothesis test results.

Hypothesis	Path	Nonstandard Coefficient	Standardization Coefficient	S.E.	C.R.	*p*
H1	PIR-APB	0.032	0.028	0.028	1.134	0.257
H2	PBPB-APB	0.470	0.572	0.021	21.899	***
H3	PDPB-APB	−0.385	−0.206	0.052	−7.470	***
H4	PIR-BWC	0.023	0.063	0.011	2.137	0.033
H5	PBPB-BWC	−0.013	−0.049	0.010	−1.306	0.192
H6	PDPB-BWC	−0.135	−0.225	0.020	−6.619	***
H7	APB-BWC	0.039	0.122	0.012	3.154	0.002

^***^p < 0.001.

As shown in Table 4, attitudes of preventive behavior did not play a mediating role between perception of infection risk and behavior of wearing condoms, but attitudes of preventive behavior played a fully mediating role (100%) between perceived benefits of preventive behavior and behavior of wearing condoms. Further, attitudes of preventive behavior played a partial mediating role between perceived disorders of preventive behavior and behavior of wearing condoms, accounting for approximately 6.9% of the total effect.

**Table 4:** Mediating effect test results.

Mediation path			Direct effect					Mediating effects					Total effects					Mediating Effect
			Percentile 95% CI					Percentile 95% CI					Percentile 95% CI					
IV	M	DV	Estimate	SE	Lower	Upper	*p*	Estimate	SE	Lower	Upper	*p*	Estimate	SE	Lower	Upper	*p*	
PIR	APB	BWC	0.063	0.035	−0.005	0.130	0.069	0.003	0.003	−0.002	0.012	0.211	0.066	0.036	−0.002	0.132	0.061	0
PBPB	APB	BWC	−0.049	0.039	−0.126	0.030	0.196	0.070	0.023	0.025	0.115	0.002	0.021	0.029	−0.035	0.077	0.488	100%
PDPB	APB	BWC	−0.225	0.036	−0.295	−0.159	0.001	−0.025	0.009	−0.044	−0.009	0.001	−0.251	0.035	−0.320	−0.185	0.001	9.96%

CI, Confidence interval; IV, Independent variable; M, Mediator; DV, Dependent variable; PIR, Perception of infection risk; PBPB, Perceived benefits of preventive behavior; PDPB, Perceived disorders of preventive behavior; APB, Attitudes of preventive behavior; BWC, Behavior of wearing condoms; SE, standard error.

## 4 Discussion

### 4.1 General sociological factors influencing condom use among sexually active college students

The use of condoms during sexual activity is among the most effective means of preventing HIV infection (31); however, various factors influence condom use among college students. In this study, we found no significant difference in behavior of wearing condoms according to gender in respondents engaged in casual sexual relationships, indicating relatively consistent protective behaviors in both men and women in this context. Although traditional beliefs may suggest that men are less willing to use condoms (32), our research findings do not support this hypothesis, indicating that gender is no longer a key factor in this behavioral choice.

No significant difference in behavior of wearing condoms score was detected between respondents undertaking junior college degrees and those studying for college degrees, suggesting that education level was not a decisive factor affecting use of condoms among college students. Further, no significant difference in protective awareness was detected between undergraduate students and those who had received higher education, which may be related to the fact that all college students receive HIV prevention and health education.

Moreover, we found no significant difference in behavior of wearing condoms on comparison of medical and non-medical students. Although medical students study more health-related courses, and theoretically have a deeper understanding of HIV and sexually transmitted infections (33), this cognitive advantage was not translated into significantly higher rates of condom use. These findings indicate that, even for college students with a medical background, behavioral changes do not occur spontaneously due to accumulation of professional knowledge, and the effectiveness of behavioral interventions requires further efforts.

Sexual orientation is an important variable affecting behavior of wearing condoms among college students. In this study, we found that the proportion of heterosexual college students using condoms during sexual intercourse was significantly higher than that of homosexual college students. This finding may be related to the fact that heterosexual individuals receive more sexual health education focused on preventing sexually transmitted diseases (such as HIV) and unintended pregnancies (34), while homosexual individuals, especially male homosexual individuals, receive less attention in traditional sex education systems and may lack systematic education on sexual health, resulting in a lower rate of safety measure implementation. In addition, trust and dependence on partners are often strong within homosexual groups (35), and individuals may reduce their use of condoms due to strong emotional trust relationships (36); however, we found no significant difference in the proportion of condom use among bisexual individuals during sexual activity relative to those in the other groups.

Commercial and casual sexual activity also had a significant impact on behavior of wearing condoms. Students who did not engage in commercial sexual activity had a significantly higher frequency of using condoms in casual relationships than those who did engage in commercial sexual activity. A higher proportion of college students who did not engage in casual sexual activity also reported using condoms, possibly due to impulsivity in temporary situations and a lack of stable emotional connections, which may lead to a failure to fully consider safety precautions.

The effect of health education on HIV infection prevention in promoting behavior of wearing condoms was also very significant. The proportion of students who had received HIV-related health education that reported using condoms during sexual activity was significantly higher than that of students who had not received such education. This finding indicates that systematic and sustained health education is crucial for enhancing awareness of protective sexual behavior among college students (37), and is an effective health intervention. The effects of health education include the improvement of knowledge. Insufficient knowledge might not be able to apply all preventive measures to reduce the spread of HIV (38). However, a study found that condom use had nothing to do with HIV knowledge (39). Therefore, the strategy of health education should be adjusted (40).

### 4.2 Impacts of perception of infection risk, perceived benefits of preventive behavior, and perceived disorders of preventive behavior on attitudes of preventive behavior

The results of this study demonstrated no significant correlation between perception of infection risk and attitudes of preventive behavior. We speculate that, when individuals are aware of the risk of HIV infection but fail to fully internalize this perception as a motivation to increase their willingness to act, or are limited by external factors, such as environment and social pressure, their attitude toward wearing condoms will not significantly improve.

Perceived benefits of preventive behavior was positively correlated with attitudes of preventive behavior. This finding suggests that, if college students can clearly understand the specific benefits of wearing condoms during sexual activity, such as effectively preventing HIV infection, reducing sexually transmitted infections, and contraception (36), their attitude toward wearing condoms during sexual activity will be more positive. Strengthening education and publicity to remind individuals that condoms are a simple, easily accessible, and efficient protective tool will help increase their rate of practical use during relevant behaviors (41).

Our research identified a negative correlation between perceived disorders of preventive behavior and attitudes of preventive behavior. Perceived behavioral disorders refer to various practical or social difficulties that individuals experience during sexual activity (23), such as the use of condoms affecting sexual pleasure, partner unwillingness to cooperate, and the inconvenience of purchasing and carrying condoms, among others (42). The stronger the perception of these obstacles, the more negative an individual’s attitude toward wearing condoms will be. This result indicates that it is not only lack of knowledge that hinders widespread condom use, but also the perception of obstacles in real life situations that significantly impact protective psychology influencing sexual behavior. Therefore, reducing perceived disorders of preventive behavior will be a key step in improving condom use, and efforts should be made to eliminate negative behavioral barriers perceived by students in their psychological and socio-cultural contexts, helping them overcome obstacles, and encouraging the use of condoms in sexual relationships.

### 4.3 Impact of perception of infection risk, perceived benefits of preventive behavior, perceived disorders of preventive behavior, and attitudes of preventive behavior on behavior of wearing condoms

Our research found that perception of infection risk was an important predictor of behavior of wearing condoms. Perception of infection risk refers to an individual’s subjective assessment of their risk of exposure to HIV infection. If individuals believe they are at a higher risk of contracting HIV, they are more inclined to take preventive measures, such as wearing condoms, during sexual activity (43). In this study, we found a significant positive correlation between perception of infection risk and behavior of wearing condoms during sexual behavior, indicating that individuals with stronger perception of infection risk were more likely to insist on condom use during sexual activity. This finding is consistent with previous research (44), indicating that increasing an individual’s perception of infection risk can effectively promote safe sexual behavior. Therefore, future health education efforts should strengthen guidance on perception of infection risk to help provide a clearer understanding of the potential health threats posed by unsafe sexual behavior.

Perceived benefits of preventive behavior is often considered a driving factor in healthy behavior patterns; however, in this study, we did not detect a significant correlation between perceived benefits of preventive behavior and behavior during sexual activity. Although many people are theoretically aware that wearing condoms can effectively prevent HIV and other sexually transmitted diseases (45), this awareness has not been directly translated into practical safety behaviors. This phenomenon may be related to the complexity of behavior. Even if individuals accept the benefits of condoms at a cognitive level, during sexual behavior, the behavioral benefits may not play a decisive role in all situations, due to various factors, such as emotions, situations, partner attitudes, or immediate decisions. Hence, simply emphasizing the benefits of behavior may not be sufficient to encourage individuals to form stable healthy behaviors, and it may be necessary to combine intervention measures with other factors, particularly in efforts to reduce individual behavioral disorders and cultivate specific attitudes.

Perceived disorders of preventive behavior has an equally important impact on behavior of wearing condoms during sexual behavior and was negatively correlated with condom use in this study. When individuals feel that there are specific obstacles influencing their behavior, they are more inclined not to use condoms. Common obstacles include the impact of condoms on sexual pleasure, partner unwillingness to cooperate, inconvenience of obtaining and using condoms, and potential social awkwardness (46). These practical and subjective barriers often lead individuals to avoid using condoms during sexual activity. Our results also indicate that, although individuals may be aware of the benefits and importance of wearing condoms, these barriers significantly weaken their willingness to engage in protective behavior. This finding emphasizes the importance of reducing behavioral disorders in future health interventions, and particularly cultivating the ability of individuals to overcome relevant barriers to ensure that they can choose to take protective measures when facing these challenges.

This study also found that attitudes of preventive behavior during sexual activity was an important factor affecting behavior of wearing condoms, and that attitudes were positively correlated with reported behavior. This finding suggests that, when individuals hold a more positive attitude toward wearing condoms, they are more likely to choose to use condoms during sexual activity. Therefore, a positive attitude can encourage individuals to use condoms during sexual activity (47, 48). This finding provides further evidence supporting the need for future prevention interventions to focus on shaping and strengthening positive attitudes toward condom use, so that individuals not only accept the benefits of condoms at a cognitive level, but also can truly put these attitudes into practice at a behavioral level.

### 4.4 The mediating role of attitudes of preventive behavior between perception of infection risk, perceived benefits of preventive behavior, perceived disorders of preventive behavior, and behavior of wearing condoms

Our analysis also showed that attitudes of preventive behavior did not play a mediating role between perception of infection risk and behavior of wearing condoms, indicating that, although individuals may have positive or negative attitudes toward the act of wearing condoms, these attitudes fail to further mediate the impact of risk perception on actual behavior in the chain of action. That is, a strong sense of risk is sufficient to prompt individuals to take action, as demonstrated in other similar studies (49), while changes in attitude do not play a decisive role.

The results of our research show that, although individuals may be aware of the many benefits of wearing condoms, this perceived benefits of preventive behavior does not directly prompt them to take safety measures during sexual activity; however, we found that attitudes of preventive behavior plays a fully mediating role between perceived benefits of preventive behavior and behavior of wearing condoms during sexual relationships. This discovery suggests that, in future health interventions, there is a need to educate individuals both about the benefits of condom use (50) and, more importantly, cultivate and enhance their positive attitudes, to ensure higher condom use rates and effectively prevent the spread of HIV.

In this study, attitudes of preventive behavior played a partial mediating role between perceived disorders of preventive behavior and behavior of wearing condoms during sexual relations, accounting for approximately 9.96% of the total effect value. Hence, although perceived disorders of preventive behavior mainly reduces condom use through direct action, a positive attitude toward condom use can alleviate the negative impact of behavioral disorders to some extent (51). For example, even if individuals perceive certain barriers to using condoms, if they have a positive attitude toward wearing condoms, they may still be able to overcome these barriers and choose to use condoms during sexual activity (52).

## 5 Conclusion

Our evidence suggests that attitudes of preventive behavior does not mediate between perception of infection risk and behavior of wearing condoms among sexually active college students, while attitudes of preventive behavior does have a partial mediating role between perceived disorders of preventive behavior and behavior of wearing condoms and plays a fully mediating role between perceived benefits of preventive behavior and behavior of wearing condoms. Therefore, in health education, it is necessary to enhance awareness of perception of infection risk, perceived benefits of preventive behavior, and attitudes of preventive behavior, as well as reducing perceived disorders of preventive behavior, thereby improving behavior of wearing condoms and reducing the risk of HIV infection among college students through sexual activity.

## 6 Limitations and outlook

This study has certain limitations that can be addressed in future research. This study focuses on the use of condoms among Chinese college students engaged in sexual activities. No attention was paid to other prevention behaviors such as voluntary counseling and testing, early diagnosis, early treatment, HIV pre exposure prevention and post exposure prevention. Although the use of condoms is one of the traditional and effective methods for preventing the spread of HIV. However, in the comprehensive intervention strategy for HIV prevention, the synergistic effect of multiple methods has played an indispensable role in reducing the risk of virus transmission and improving the health level of the population. Pre exposure prophylaxis (PrEP) and post exposure prophylaxis (PEP), which involve the use of antiviral drugs in advance, can significantly reduce the risk of HIV infection. Voluntary Counseling and Testing (VCT) can detect HIV infected individuals early, promote early diagnosis and treatment, and reduce virus transmission. Future research will focus on voluntary counseling and testing, early diagnosis, early treatment, pre exposure prevention and post exposure prevention of HIV.

## Data Availability

The raw data supporting the conclusions of this article will be made available by the authors, without undue reservation.
