# Supplementary material for: A cross-sectional survey about behavior of wearing condoms among college students who engage in sexual activity: the mediating role of attitudes of preventive behavior
Source: Front Public Health. 2025 May 23;13:1544564. doi: 10.3389/fpubh.2025.1544564 (PMC12141243; doi:10.3389/fpubh.2025.1544564)
Supplement: Supplementary file 1 [file Table_1.docx]

Table S1. PIR, PBPB, PDPB, and APB.

| Research Constructs | Measurement Items |
| --- | --- |
| PIR | 1. The person I have sexual relations with may be at risk of contracting HIV. 2. Behaviors without condoms are more likely to be infected with HIV. 3. After being infected with HIV, it will affect my physical, psychological, economic, and social activities. |
| PBPB | 1. Wearing condoms during sexual activity can prevent HIV infection. 2. Wearing condoms during sexual activity will make me and my sexual partner feel more at ease. 3. Wearing condoms during sexual activity will benefit me and my sexual partner. |
| PDPB | 1. Wearing condoms during sexual activity can affect my relationship with my sexual partner. 2. Wearing condoms during sexual activity can affect the pleasure of sexual activity. 3. Buying condoms can make one feel embarrassed if discovered by others. 4. Using condoms can have side effects. |
| APB | 1. When engaging in sexual activity without intending to conceive, condoms should be used. 2. I am willing to use condoms during sexual activity. 3. When engaging in sexual activity, I will request the use of condoms. 4. I can overcome obstacles and use condoms during sexual activity. 5. When engaging in sexual activity, I will request to wear a condom. If I am unable to wear a condom, I will promptly terminate the sexual activity to ensure my safety. |
